# Proctored Step by Step Training Program for GreenLight Laser Anatomic Photovaporization of the Prostate: A Single Surgeon's Experience

**DOI:** 10.3389/fsurg.2021.705105

**Published:** 2021-07-29

**Authors:** Francesco Sessa, Riccardo Campi, Stefano Granieri, Agostino Tuccio, Paolo Polverino, Pietro Spatafora, Arcangelo Sebastianelli, Andrea Cocci, Anna Rivetti, Mauro Gacci, Marco Carini, Sergio Serni, Rino Oriti, Andrea Minervini

**Affiliations:** ^1^Unit of Urological Robotic Surgery and Renal Transplantation, Careggi Hospital, University of Florence, Florence, Italy; ^2^Department of Experimental and Clinical Medicine, University of Florence, Florence, Italy; ^3^Azienda Socio-Sanitaria Territoriale (ASST)-Brianza, General Surgery Unit, Vimercate, Italy; ^4^Unit of Urological Oncologic Minimally Invasive Robotic Surgery and Andrology, Careggi Hospital, University of Florence, Florence, Italy; ^5^Unit of Urology, S.Stefano Hospital, University of Florence, Prato, Italy

**Keywords:** GreenLight laser, modular training, benign prostate obstruction, lower urinary tract symptoms, photovaporization of the prostate

## Abstract

**Objectives:** To evaluate the feasibility and safety of a proctored step-by-step training program for GreenLight laser anatomic photovaporization (aPVP) of the prostate.

**Methods:** Data from patients undergoing aPVP between January 2019 and December 2020 operated by a single surgeon following a dedicated step-by-step proctored program were prospectively collected. The procedure was divided into five modular steps of increasing complexity. Preoperative patients' data as well as total operative time, energy delivered on the prostate and postoperative data, were recorded. Then, we assessed how the overall amount of energy delivered and the operative times varied during the training program. Surgical steps were analyzed by cumulative summation. Univariable and multivariable regression models were built to assess the predictors of the amount of energy delivered on the prostate.

**Results:** Sixty consecutive patients were included in the analysis. Median prostate volume was 56.5 mL. The training program was succesfully completed with no intraoperative or meaningful post-operative complications. The energy delivered reached the plateau after the 40th case. At multivariable analysis, increasing surgeon experience was associated with lower amounts of energy delivered as well as lower operative times.

**Conclusions:** A step-by-step aPVP training program can be safely performed by surgeons with prior endoscopic experience if mentored by a skilled proctor. Considering the energy delivered as an efficacy surrogate metrics (given its potential impact on persistent postoperative LUTS), 40 cases are needed to reach a plateau for aPVP proficiency. Further studies are needed to assess the safety of our step-by-step training modular program in other clinical contexts.

## Introduction

Lower urinary tract symptoms (LUTS) due to benign prostate hyperplasia (BPH) are a common complaint in adult men with a major impact on quality of life (QoL) (1–3). Nowadays, the most relevant surgical options for benign prostate obstruction (BPO) include simple prostatectomy, trans-urethral resection of the prostate (TURP), laser enucleation of the prostate (i.e., HoLEP/ThuLEP, etc.), and Greenlight laser vaporization of the prostate (4–8).

While surgical management of LUTS due to BPO has evolved toward the concept of minimally-invasive surgery and will likely replace open surgery in the next years (9, 10), there are currently no structured validated training curricula for endoscopic treatment of BPO.

To a great extent, urology is at the forefront of minimal invasive surgery and its procedures might have a steep initial learning curve. As such, given the impact of the surgeon's experience on variability in perioperative outcomes after urological surgery, training and assessment methods in surgical specialties become a clinical priority, as demonstrated by the continuous need for clear structure and reproducible models present in different urological fields (10–12).

Despite GreenLight laser photovaporization (PVP) having been standardized and described at all levels, from standard PVP to more complex procedures involving enucleation (13, 14), there is a lack of studies evaluating how to safely train novice surgeons with prior endoscopic experience in PVP using robust and standardized metrics. Moreover, while several studies aimed at evaluating the safety and efficacy of PVP focused the analysis on the number of procedures required in order to achieve proficiency (15–17), data are limited by the heterogeneous criteria used to assess the learning curve (18–21).

As such, evaluating novel frameworks to develop step-by-step proctored training programs in PVP is key, given the potential impact of the surgeon's experience on postoperative outcomes, such as the persistence of burdensome lower urinary tract symptoms (LUTS) impacting on patients' quality of life (22–24). In fact, previous studies confimed an association between surgery-related variables, including the amount of energy delivered on the prostatic tissue, and postoperative LUTS (25, 26).

In this study we evaluated the feasibility and safety of a proctored step-by-step training program for GreenLight laser anatomic photovaporization of the prostate (aPVP), focusing on the variation of the energy delivered on the prostate and the overall operative time as efficacy surrogate metrics.

## Materials and Methods

### Design, Setting, and Partecipants

After institutional Ethical Committee and review board approval, all data from consecutive patients who underwent aPVP with 180-W XPS Green Light laser at an Academic tertiary referral Center between January 2019 and December 2020 were prospectively collected. All procedures were performed by a single surgeon (F.S.) with prior experience with TURP (*n* = 120) and no experience in aPVP, and under the guidance of an experienced proctor (R.O.) (>500 aPVP).

### Step-By-Step Training Program

Our proctored step-by-step training program included the following consecutive phases:

a) Careful definition of “modular” surgical steps of aPVP, based on a comprehensive review of surgical videos in collaboration with the proctor to progressively acquire the theoretical knowledge on minimally-invasive treatment for BPH.
b) Real-case observation in the operating theater of at least 50 aPVPs performed by the proctor
c) Modular step-by-step surgical training in the operating theater, under the guidance of the proctor, in order to progressively acquire the practical skills and technical nuances related to each step of the procedure, as previously reported in other urological settings (27–29). Specifically, the surgeon performed aPVP using a 180-W GreenLight XPS device, following established technical principles (30). The proctor supervised all the procedures, taking over the control of surgery in case the surgeon in training was not able to safely conclude the surgery.

All patients received antibiotic prophylaxis and were discharged without a catheter after an ultrasound post-residual volume (PVR) assessment.

### Surgical Steps

**Figure 1:**
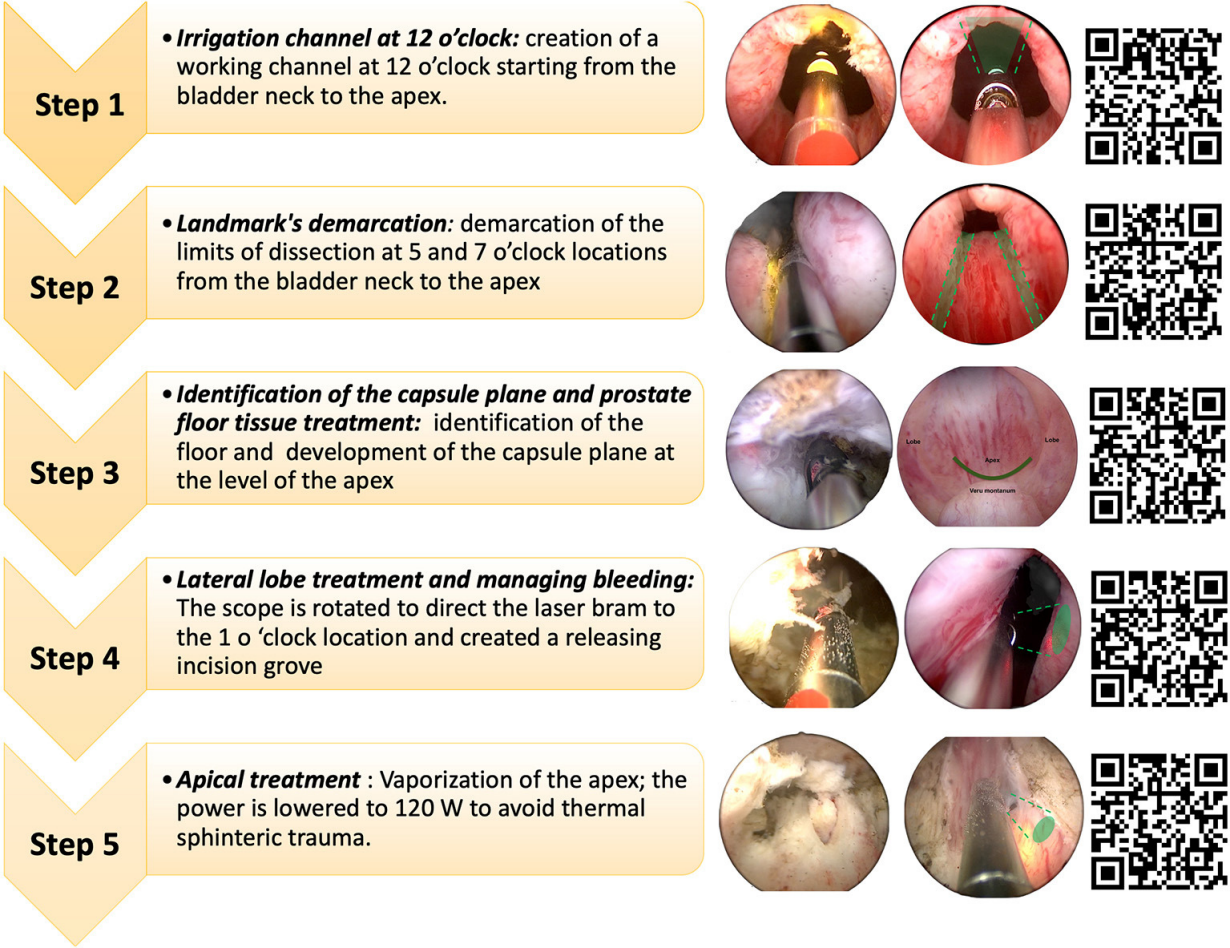
Surgical steps of anatomic photopvaporization of the prostate (aPVP).

The surgical procedure was divided in a step-by-step fashion (Figure 1). Specifically, the program included:

Step 1: *creation of an irrigation channel*: creation of a working channel at 12 o' clock starting from the bladder neck to the apex. The appropriacy of the procedure included a continuous rotation of the fiber as well as the manteinance of 1–3 mm working distance.
Step 2: *landmark demarcation*: demarcation of the limits of dissection at 5 and 7 o' clock from the bladder neck to the apex using the 180 W power setting. This maneuver allows for a visual guide to avoid exceding beyond the verumontanum.
Step 3: *prostate floor tissue treatment*: once the initial groves are made, the next step is to identify the floor and to develop the capsule plane at the level of the apex. In this phase, the key point of this step is to treat tissue with the laser fiber cap in contact with the capsule and rotating the cap and delivering energy horizontally along-side the capsular fibers. This permits to reduce the risk of capsular perforation and urinary irritative symptoms.
Step 4: *lateral lobe treatment and managing bleeding:* The scope is rotated to direct the laser bram toward 1 o ‘clock and to create a releasing incision grove, respecting the following anatomical landmarks: prostate capsule, baldder neck, and verumontanum.
Step 5: *apical treatment* the power is lowered to 120 W to avoid thermal sphinteric trauma.

### Outcome Measures and Statistical Analysis

Surgeon experience was analyzed as a continuous variable (number of consecutive procedures). The following data were included in the database: patient age, body mass index (BMI), anticoagulant and antiplatelet therapy at surgery, American Society of Anesthesiologists (ASA) score, Charlson comorbidity index (CCI), anticoagulant/antiplatelet medication, previous BPH medical history, history of retention, preoperative prostate volume and PRV (as recorded by a single radiologist). Preoperative symptoms index score including international index of erectile function (IIEF 5), Quality of life (QoL), overactive bladder short form (OABQ-SF), international consultation on incontinence questionnaire-urinary Incontinence short form (ICIQ-UI) preoperative International Prostate Symptom Score (IPSS), prostate volume (PV), prostate-specific antigen (PSA) level, maximum flow (Qmax) average flow (Qmed), and postoperative PVR (as assessed by a single urologist) were also recorded. Patients with preoperative prostate volumes >100 mL were excluded. In addition, the operative time and energy delivered on the prostatic tissue (kJ/mL) were assessed for each surgical step of the procedure. Intraoperative and early (30-d) and 6-months post-operative complications as well as functional outcomes (ΔIPSS,ΔIIEF, ΔQoL, ΔOABQ-SF, ΔICIQ-SF) were collected prospectively.

Given the relatively small sample size of our series, and the low number of events (i.e., complications and adverse functional outcomes), we could not evaluate the efficacy of the training program with regard to established composite metrics. As such, to evaluate the proficiency of the surgeon in training over time, we relied on the amount of energy delivered on the prostate as a reference variable to benchmark surgical performance. The rationale for choosing such primary outcome relies in its potential association with postoperative storage LUTS (25, 26). In this regard, our aim was to provide a safe framework to allow a surgeon with prior endoscopic experience to follow a step-by-step training program in aPVP under the guidance of a proctor, maximizing patient outcomes and keeping the risk of adverse events to a minimum (22–24). To do so, we also identified the breakpoint at which the slope of the “learning curve” regarding the energy delivered became flat.

Descriptive statistics were obtained reporting medians and inter-quartile ranges (IQR) for continuous variables and number and percentages for categorical variables, as appropriate, were obtained.

All surgical steps were analyzed by the cumulative summation (CUSUM) method, which recognizes the importance of time and experience in clinical practice. Competency of the procedure was defined as the first turning point of the curve plateau, and proficiency was defined as the turning point at which the slope of the curve becomes less steep. Furthermore, Shewhart control charts for one-at-time data were built to evaluate how the group summary statistics deviated above or below the process center (+2SD = alert line, +3SD = alarm line).

Linear regression analysis was performed to assess the association between surgeon experience (number of consecutive procedures) and the amount of energy delivered on the prostate. The association between the energy delivered and persistent postoperative symptoms (Δ-IPSS, IIEF, QoL, OABQ-SF, ICIQ-SF) was assessed using the Pearson correlation. All tests were two-sided with a significance level set at *p* < 0.05.

Linear regression was performed with a simple linear model to estimate the relationship between the number of procedures and the surgeon improvement in terms of energy delivered. Cubic smoothing spline was used to graphically explore the relationship between surgical experience and the aforementioned dependent variables. The influence of different variables on the outcomes was assessed by building two multivariate regression models, one per outcome variable, providing adjusted odds ratios, and 95% confidence intervals (CI). All variables not contributing to the model (overfitting) were removed one-by-one from the model at each step based on the *R*^2^ value. Multicollinearity was preventively assessed by examining the variance inflation factor (VIF).

The same analyses were used to evaluate the association between surgeon experience and overall operative time for aPVP.

Data were recorded in a computerized spreadsheet (Microsoft Excel 2016; Microsoft Corporation, Redmond, WA, USA). The statistical analysis was conducted using SPSS (IBM Corp. Released 2017. IBM SPSS Statistics for Windows, Version 25.0. Armonk, NY, USA) and R (The Comprehensive R Archive Network—CRAN, ver. 4.0.0 x64), using “cusum” and “qcc” packages.

## Results

Overall, 70 consecutive patients were included in the study. Of these, the first 10 cases were excluded from the analysis as they were treated with standard PVP rather than pure aPVP. As such, 60 consecutive patients were included in the analytic cohort. Median follow-up was 12 months (IQR 10-13).

### Preoperative and Intraoperative Features

Patients' characteristics, including age, BMI, CCI, and ASA score are reported in Table 1. The proportion of patients receiving oral anticoagulation was 28 and 6% for anticoagulant and antiaggregant therapy, respectively. Overall, median (IQR) prostate volume was 56.5 mL (43.5–78.2), Qmax 10 mL/s (8.2–11.7), preoperative PVR 82.5 mL (60.0–127.5). Median IPSS preoprative was 25 (21.2–30), OABQ-SF 59.5 (50.2–65), ICIQ-SF 0 (0-1), IIEF-5 21(16.2–24) and QoL score 4 (4–5).

**Table 1:** Preoperative characteristics of patients.

**Variables**	**Median (IQR)**
Age, years	65 (62–70)
BMI, kg/m^2^	26 (24–27)
Qmax, mL/s	10 (8.25–11.75)
PVR, mL	82.50 (60–127.50)
PSA, ng/mL	2.90 (2.02–3.89)
IPSS	25 (21.25–30)
QoL	4 (4–5)
OABQ-SF	59.50 (50.25–65)
ICIQ-SF	0 (0–1)
IIEF-5	21 (16.25–24)
Prostate volume, mL	56.50 (43.50–78.25)

*BMI, Body mass index; Qmax, maximum flow; PSA, prostate-specific antigen; PVR, post-void residual; IPSS, International Prostate Symptom Score, QoL, Quality of Life; OABQ-SF, Overactive Bladder Short Form; ICIQ-SF, International Consultation on Incontinence Questionnaire-Short Form; IIEF-5, International Index of Erectile Function*.

Overall, the median operative time was 43 min (IQR 38.2–52.2) with a median energy delivered of 2,387 kJ/mL. An overview of the operative time and energy delivered for each step of aPVP and for the whole procedure is depicted in Supplementary Table 1.

### Perioperative and Postoperative Features

Generally, no intraoperative complications were recorded. Two patients (2/70, 3%) experienced early postoperative complications (one patient with clots retention requiring blood transfusion and one with postoperative acute urinary retention requiring catheterization). Median catheterization and hospitalization time was 2 days (IQR 2-3).

Functional outcomes 6 months after surgery are shown in Supplementary Table 2.

Median ΔHb, ΔPSA, ΔQmax was 0,60 g/dl, 1.56 ng/mL, 18.5 mL/s, respectively.

Comprehensively, we recorded a meaningful post-operative improvement in both storage and functional symptoms as well as in global quality of life scores at validated questionnaries (median ΔIPSS, Δ OABQ-SF, ΔICIQ-SF, ΔQoL were 15.5, 28, 0, 3, respectively).

A significant inverse correlation between the amount of energy delivered on the prostatic tissue and the degree of improvement of LUTS (as assessed by ΔOABQ-SF and ΔIPSS) was detected considering the entire surgical procedure. The same analysis applied to the individual steps of the aPVP procedure (Figure 1) confirmed a significant inverse correlation for all steps regarding the general improvement in IPSS, while only for steps 1-3 regarding the imporvement in storage LUTS (as assessed by ΔOABQ-SF) (all *p* < 0.05; data not shown).

### Evolution of Intraoperative Parameters Over the Training Period

Control charts for total and step-specific energy delivered on the prostate during aPVP are showed in Supplementary Figure 1. The punctual distribution depicted a trend toward lower amounts of energy delivered for increasing surgeon's experience.

**Figure 2:**
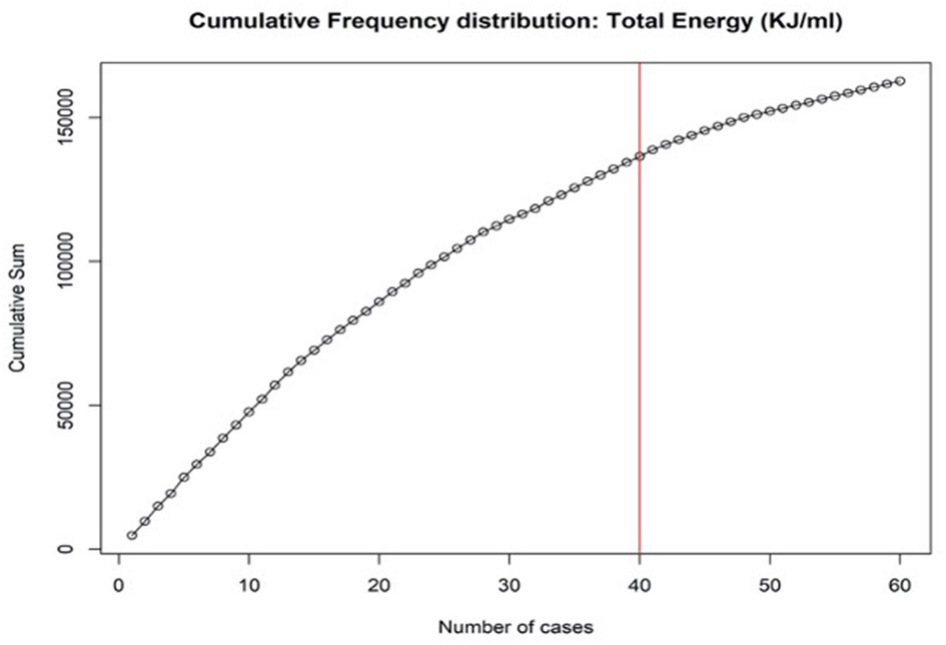
Cumulative summation analysis of total energy per ml of prostate volume during the training process.

**Figure 3:**
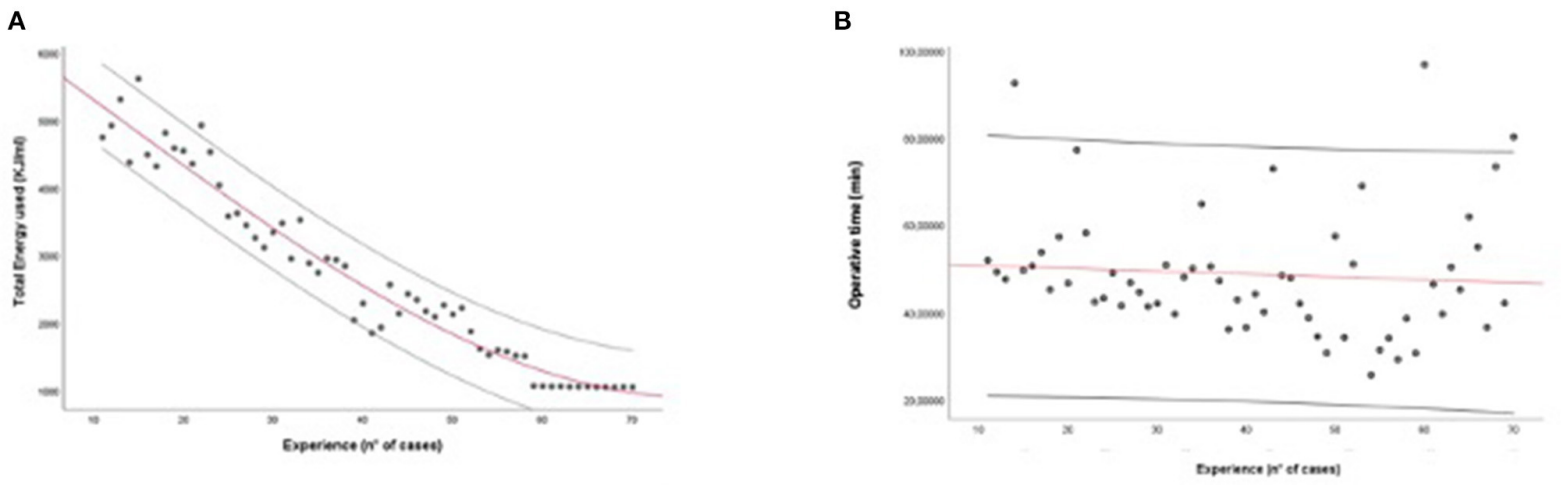
**(A,B)** Linear regression: Total energy adopted/number of procedures **(A)** and total operative time/number of procedures **(B)** during the training process.

During the training period, a progressive linear drop in energy delivered on the prostate was observed. At CUSUM analisys the surgeon's proficiency reached a plateau after the 40th case, as reported in Figure 2. The same analysis stratified by the steps of the procedure showed that the plateau was reached after 37 cases for step 1, 28 cases for step 2, 41 cases for step 3, 39 cases for step 4, and 42 cases for step 5 (Supplementary Figures 2A–E). The analysis of the distribution of the total energy delivered on the prostatic tissue over the consecutive cases confirmed a progressive decrease in the energy delivered over the training period (Figure 3A). A similar trend was noted for the overall operative time, which progressively decreased over the training period (Figure 3B).

At univariable analysis, increasing ASA score (*p* = 0.02), CCI (*p* = 0.006), need for BPH therapy (*p* < 0.001), increasing preoperative prostate volume (*p* < 0.001) were significantly associated to the total amount of energy delivered to the prostate during aPVP. On the contrary, increasing surgeon's experience (number of consecutive case) was inversely associated with the amount of energy delivered (*p* < 0.001). At Multivariable analysis, surgeon's experience remained the only independent predictor of the energy delieverd on the (*p* = 0.001) (Supplementary Table 3).

Surgeon's experience (number of consecutive case) and preoperative prostate volume were found to be the only indepenedent predictors of the overall operative time at multivariable analysis (data not shown).

## Discussion

In this study we assessed the feasibility and safety of a proctored step-by-step training program for GreenLight laser anatomic photovaporization of the prostate for a novice surgeon with prior experience in TURP, using reliable surrogate metrics to evaluate the efficacy of surgery and the trainer's proficency over time.

Given the increasing interest in standardization of training programs for minimally-invasive surgery in Urology (22, 23), as well as the potential impact of surgeon's experience on postoperative adverse outcomes in the specific setting of PVP (25, 26), our study fills a priority gap in the literature by proposing a framework to allow surgeons with basic endoscopic experience to gain proficiency in a more complex procedure such as aPVP. In this regard, our study provides several key findings to contextualize the role of proctored training programs for minimally-invasive treatment of BPH.

The first key finding of the study is that a step-by-step training program for aPVP is feasible and safe for a surgeon with no prior experience in endoscopic enucleation of the prostate if properly mentored. In fact, our program allowed the surgeon in training to achieve proficiency while ensuring optimal patient outcomes. Importantly, while previous studies evaluating the learning curve of Greenlight vaporization/enucleation of the prostate relied on stronger endpoints such as surgical complications to benchmark surgical performance (17–21), we could not use such outcomes in our study due to the relatively low sample size and low number of events. As such, we relied on the amount of energy delivered on the prostate as a potential surrogate of the efficacy of surgery, being associated with the risk of persistent LUTS in the postoperative period (25, 26). As shown in Figures 2, 3, a progressive reduction of the amount of energy delivered on the prostate was recorded with increasing surgeon's experience; of note, in our study as well as in previous experiences, the amount of energy delivered was associated with the degree of persistent burdensome LUTS after surgery (25, 26). Our analysis higlighted that the amount of energy delivered on the prostate plateud after 40 cases (Figure 2). Interestingly, the results of multivariable analysis point to surgeon's experience as a key determinant of the amount of energy delivered on the prostate as well as the overall operative time.

While several studies have previously evaluated the learning curve for holmium laser enucleation of the prostate (HoLEP) (30), only few experiences have been published so far on PVP (17–21, 30, 31). In this scenario, our study confirms the opportunity to achieve favorable outcomes even by less experienced surgeons (19), if appropriately mentored through a structured step-by-step program.

In this regard, future efforts should be focused on implementation of such training pathway through the integration of simulation-based exercises specifically designed for PVP (32, 33).

Lastly, a key finding of the study is that, at CUSUM analysis, the steps requiring more cases to achieve proficiency by the surgeon in training in terms of energy delivered were the 4 and 5th steps, suggesting that such critical steps are those requiring a more careful mentoring and monitoring of the surgeon's technical performance to ensure optimal patient outcomes during the training period. From a surgical perspective, this finding might be explained by a higher degree of difficulty in respecting the anatomical landmarks (including prostate capsule, bladder neck, and verumontanum) during the lateral lobe treatment, as well as the need to avoid thermal sphinteric trauma during apical treatment.

Despite its novelty, our study is not devoid of limitations. First, the step-by-step training program (including the observation of 50 PVPs before startuing the training and the division of the procedure into 5 steps) was designed according to arbitrary criteria; yet, there are currently no validated frameworks to evaluate the learning curve of aPVP so far. Second, the analysis of the training process was performed on a single surgeon's experience, potentially limiting the reproducibility of our findings. Third, the study included a relatively low number of cases, and selection bias cannot be entirely ruled out. For instance, our findings cannot be applied to patients with preoperative prostate volumes >100 mL. As such, further studies are needed to confirm our findings in patients with larger prostates. Lastly, the presence of a proctor mentoring all steps of the procedure during the training process might have positively influenced the final perioperative outcomes (i.e., low complication rate despite the step-by-step program). However, this was intentionally pursued to ensure the best outcomes for the patients regardless of the surgeon's learning curve.

Acknowledging these limitations, our study provides a first reliable and feasible framework to standardize the training of aPVP for surgeons with prior exposure to TURP but no experience in endoscopic enucleation of the prostate, and might prompt the development of codified modular training curricula for aPVP and other endoscopic techniques in the future.

## Data Availability Statement

The original contributions generated for this study are included in the article/Supplementary Material, further inquiries can be directed to the corresponding author/s.

## Ethics Statement

Ethical review and approval was not required for the study on human participants in accordance with the local legislation and institutional requirements. The patients/participants provided their written informed consent to participate in this study.

## Author Contributions

FS, RO, and AT: conception and design. SG: statistical analysis. RC and SG: analysis and interpretation of data. PP and AR: acquisition of data. PS, AS, and FS: drafting of the manuscript. AC, MG, and AM: critical revision of the manuscript. MC, SS, and AM: supervision. All authors contributed to the article and approved the submitted version.

## Conflict of Interest

The authors declare that the research was conducted in the absence of any commercial or financial relationships that could be construed as a potential conflict of interest.

## Publisher's Note

All claims expressed in this article are solely those of the authors and do not necessarily represent those of their affiliated organizations, or those of the publisher, the editors and the reviewers. Any product that may be evaluated in this article, or claim that may be made by its manufacturer, is not guaranteed or endorsed by the publisher.

## Supplementary Material

Supplementary Figure 1 **(A–F)** Swehchart charts for total **(A)** and step-specific **(B–F)** KJ/mL used during the training process.

Supplementary Figure 2 **(A–E)** Cumulative summation analysis of total energy per ml of prostate volume during the training process for each step of Greenlight aPVP.

Supplementary Table 1 Intraoperative data, overall and stratified by surgical step.

Supplementary Table 2 Postoperative data.

Supplementary Table 3 Uni and Multivariate regression model for energy delivered on the prostate.
