# Supplementary figures and images for: Proctored Step by Step Training Program for GreenLight Laser Anatomic Photovaporization of the Prostate: A Single Surgeon's Experience
Source: Front Surg. 2021 Jul 29;8:705105. doi: 10.3389/fsurg.2021.705105 (PMC8358301; doi:10.3389/fsurg.2021.705105)

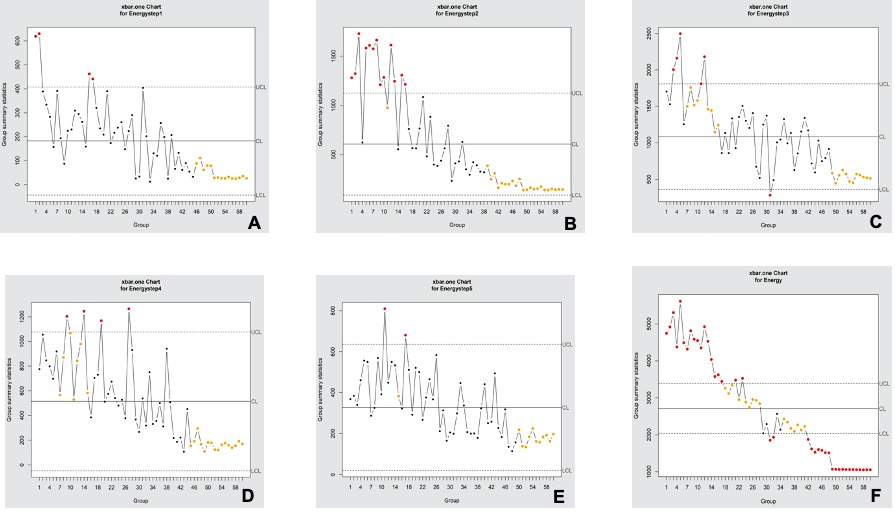

Supplement: Supplementary Figure 1 — (A–F) Swehchart charts for total (A) and step-specific (B–F) KJ/mL used during the training process. [file Image_1.JPEG]

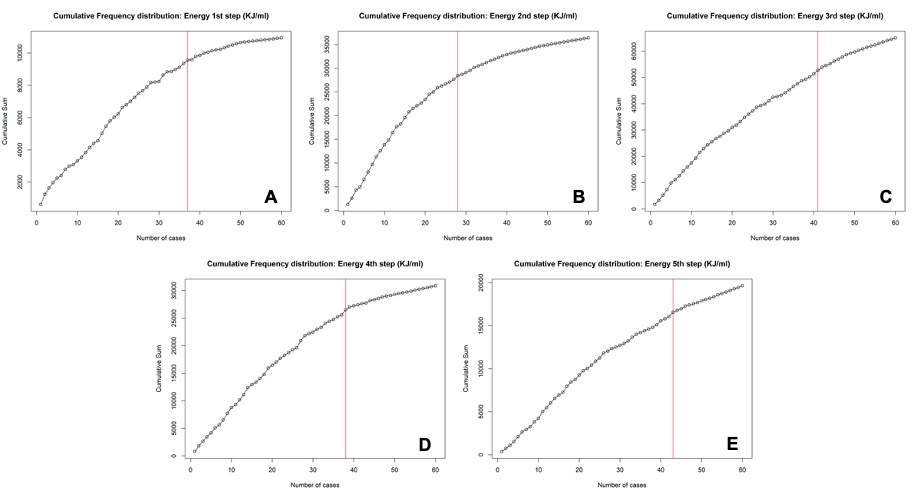

Supplement: Supplementary Figure 2 — (A–E) Cumulative summation analysis of total energy per ml of prostate volume during the training process for each step of Greenlight aPVP. [file Image_2.JPEG]
