# Supplementary material for: Proctored Step by Step Training Program for GreenLight Laser Anatomic Photovaporization of the Prostate: A Single Surgeon's Experience
Source: Front Surg. 2021 Jul 29;8:705105. doi: 10.3389/fsurg.2021.705105 (PMC8358301; doi:10.3389/fsurg.2021.705105)
Supplement: Supplementary Table 1 — Intraoperative data, overall and stratified by surgical step. [file Table_1.docx]

**Supplementary Table 1. Intraoperative data, overall and stratified by surgical step**

|  | **Energy/volume of tissue**, kJ/mL | **Time**, min |
| --- | --- | --- |
| **Overall**, (median; IQR) | 2387 (1541.50-3613.75) | 45 (38.25-52.25) |
| **Step 1**, (median; IQR) | 158.50 (40.75-261.75) | 5 (3-6) |
| **Step 2**, (median; IQR) | 404 (198.25-952) | 10 (8-13) |
| **Step 3**, (median; IQR) | 1088.50 (602-1397.25) | 14 (12-17) |
| **Step 4**, (median; IQR) | 491 (188-769) | 9 (7-12) |
| **Step 5**, (median; IQR) | 305.50 (197.50-447.50) | 7 (5-9.75) |

**Supplementary Table 2. Postoperative data**

| **Patients** | **Median (IQR)** |
| --- | --- |
| **Catheterization time**, days | 2 (2-3) |
| **Hospitalization time**, days | 2 (2-2) |
| **Δ Qmax**, mL/s | 18.50 (14-22) |
| **Δ Hb**, g/dL | 0.60 (0.30-0.87) |
| **Δ PSA**, ng/mL | 1.56 (0.70-2.29) |
| **Δ IPSS** | 15.50 (12-20) |
| **Δ QoL** | 3 (2-4) |
| **Δ OABQ-SF** | 28 (17.25-31) |
| **Δ ICIQ-SF** | 0 (0-0) |
| **Δ IIEF-5** | 0 (0-0) |

Qmax: maximum flow; Hb: haemoglobin; PSA: prostate-specific antigen; IPSS:International prostate symptom score; QoL: Quality of life; OABQ-SF: overactive bladder short form; ICIQ-SF: international consultation on incontinence questionnaire short form; IIEF-5 international index of erectile function;

**Supplementary Table 3. Uni and Multivariate regression model for energy delivered on the prostate.**

| **Variable** | **Univariate analysis** | | | | | **Multivariate analysis** | | | | |
| --- | --- | --- | --- | --- | --- | --- | --- | --- | --- | --- |
|  | **Standardized Beta** | **p** | **95% CI** | | **Standardized Beta** | | **p** | **95% CI** | |  |
|  |  |  | **Lower bound** | **Upper bound** |  |  |  | **Lower bound** | **Upper bound** |  |
| **Number of consecutive cases** | -0,966 | < 0.001 | -79,57 | -69,14 | -0,973 | | < 0.001 | -81,97 | -67,84 |  |
| **ASA score** | 0,292 | 0,024 | 110,10 | 1488,51 | -0,032 | | 0,437 | -313,93 | 137,62 |  |
| **Age adjusted CCI score** | 0,353 | 0,006 | 146,13 | 820,38 | 0,033 | | 0,422 | -65,83 | 155,02 |  |
| **BPH therapy** | -0,449 | < 0.001 | -519,64 | -162,45 | -0,01 | | 0,798 | -67,48 | 52,11 |  |
| **Preoperative prostate volume (mL)** | 0,475 | < 0.001 | 11,27 | 36,62 | 0,025 | | 0,557 | -2,71 | 4,97 |  |

ASA: American Society of Anesthesiologists; BPH: Benign prostate hypertrophy; CCI: Charlson Comorbidity Index
